# Extra-Axial Dural-based Cavernous Hemangioma of Skull Base in a 15-year-old Male: A tale of complex case with favorable outcome

**DOI:** 10.12669/pjms.41.13(PINS-NNOS).13502

**Published:** 2025-12

**Authors:** Zubair Mustafa Khan, Haseeb Mehmood Qadri, Khawar Anwar, Faiqa Ijaz Khan

**Affiliations:** 1 Zubair Mustafa Khan, MBBS, FCPS, Punjab Institute of Neurosciences, Lahore, Pakistan; 2 Haseeb Mehmood Qadri, MBBS, Punjab Institute of Neurosciences, Lahore, Pakistan; 3 Khawar Anwar, MBBS, MS, Punjab Institute of Neurosciences, Lahore, Pakistan; 4 Faiqa Ijaz Khan, MBBS, Punjab Institute of Neurosciences, Lahore, Pakistan

**Keywords:** Brain cavernous hemangiomas, Cavernous hemangiomas, Cavernous angiomas, Middle cranial fossa, Pakistan, Cerebral cavernous hemangiomas

## Abstract

Middle cranial fossa hemangiomas are challenging owing to the complex vascular anatomy of the region. Only few cases have been reported to date, in our setting. A 15-year-old male presented with the headache, right sided visual deterioration for 18 months. He had right pupil dilated, non-reactive, ptosis and ophthalmoplegia. Magnetic resonance imaging (MRI) revealed right middle fossa heterogeneous enhancing lesion. On angiography, internal carotid artery (ICA) showed cavernous segment blush. Digital subtraction angiography was suggestive of diminutive right ICA; no distal filling beyond its cavernous segment. Patient was re-operated, excision was done. Intraoperatively lesion was extra-axial and highly vascular. Histopathology was conclusive of OLIG2 and SSTR2 negative hemangioma. Post-operative recovery was uneventful. While probing the differentials of skull base lesions, especially near ICA, cavernous sinus hemangiomas must be considered. Although extremely rare but the vascular nature poses a threat to life, if not meticulously investigated and operated timely.

## INTRODUCTION

Cerebral cavernous malformations has incidence of 2-3%, with no gender preference.^1^,^2^ They can be sporadic or familial, later being 40-60% of cases, usually autosomal dominant inheritance pattern.^1^,^2^ Intracranial cavernous hemangiomas are highly vascular but benign lesion.^3^ Usually presents with the symptoms of cranial nerve palsies, neurological deficit, and hemorrhage or fits.^4^-^6^ They can be either intra or extra axial, later has exceptionally low prevalence.^7^,^8^ They have predilection towards cerebellopontine angle and middle cranial fossa.^7^

Extra axial dural based cavernous hemangiomas are classified in two groups. First originating from sellar para-sellar space mostly called cavernous. Second convexities, falx, skull base or cranial nerves.^9^ Thus they can mimic meningioma and thus have chance of being misdiagnosed.^10^ They also resembles metastases, arteriovenous malformation (AVM), schawannomas, lymphomas, teratomas, sarcoid granuloma, or chondrosarcomas on imaging, thus a keen scrutinization of imaging in required pre-operatively.^3^,^10^,^11^ Extra-axial cavernous angiomas can be dural based or parenchymal based.^12^ Clinical presentation, imaging features and surgical approaches are different for both.^10^ However they both have single layered endothelium lined vascular space along with elastic tissue.^11^ Because of high vascularity and critical anatomical location there is postoperative risk of bleeding and neurological deficit.^11^ We report a unique case of extra-axial cavernous hemangioma of middle cranial fossa. Which needed a dedicated and expert neurosurgical care for its management.

## CASE PRESENTATION

A fully conscious 15-year-old male presented in April 2024, with history of mild headache, right side decrease vision and drooping of right eyelid, symptoms were progressive over the period of 18 months. He underwent right frontal craniectomy from a private setup ten days ago for right temporal space occupying lesion (SOL). However, the biopsy of the lesion was inconclusive and he had no investigations available.

Upon examination at our clinic, his right pupil was dilated and non-reactive to light. On examination, he had complete ophthalmoplegia in right eye. However, the light perception and finger counting were present. Left eye examination was completely normal. He also complained of right-sided facial numbness off and on, but trigeminal nerve examination was unremarkable at that time. There were no other significant neurological findings on examination. He had no comorbidities and his family history was unremarkable. Differential diagnoses of sphenoid wing meningiomas, schawannomas, parasellar SOL and caraniopharyngiomas were made.

**Fig.1:**
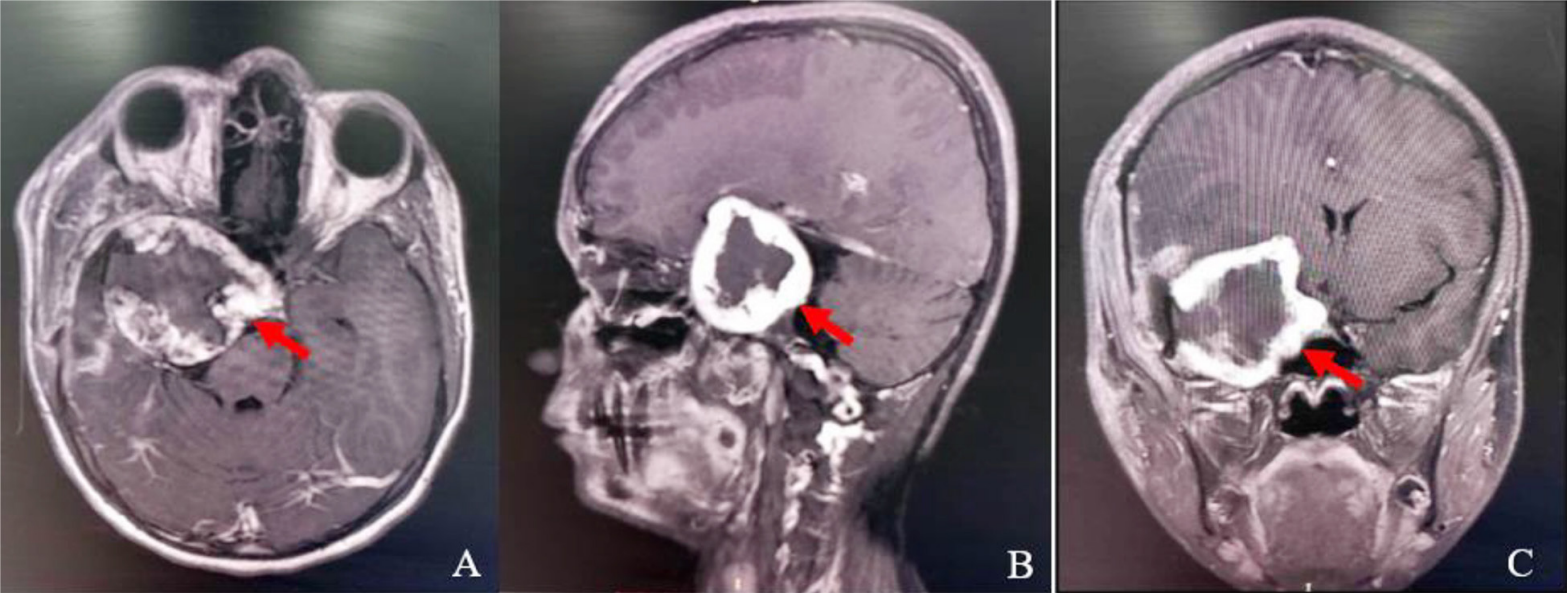
**A:** MRI brain with IV contrast showing peripherally contrast enhancing right extra-axial space occupying lesion (represented by red arrow). **B:** Sagittal view. **C:** Coronal view.

**Fig.2:**
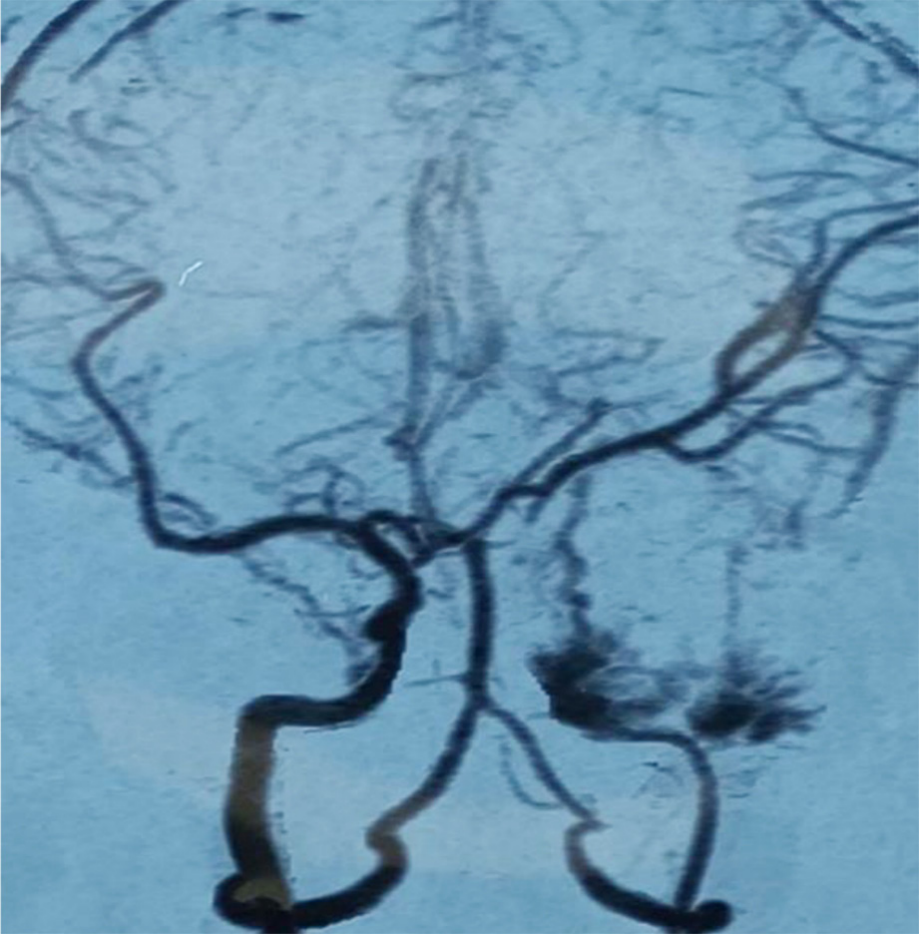
CT angiogram (CTA) showing blush after contrast injection in right internal carotid artery (ICA).

Contrast-enhanced magnetic resonance imaging (CE-MRI) showed an extra-axial, well-circumscribed, peripherally heterogeneous contrast enhancing lesion; hypointense on T1 and hyperintense on T2, in right temporal and parasellar region with hypointense center and small cystic component towards the temporal bone (Fig.1). Multiple flow void signals were well-appreciated. Computerized tomographic angiography (CTA) revealed hypoplastic right ICA with blush in its cavernous segment (Fig.2). In the light of imaging differentials narrowed down to vascular malformation, hemangioma and meningioma.

**Fig.3:**
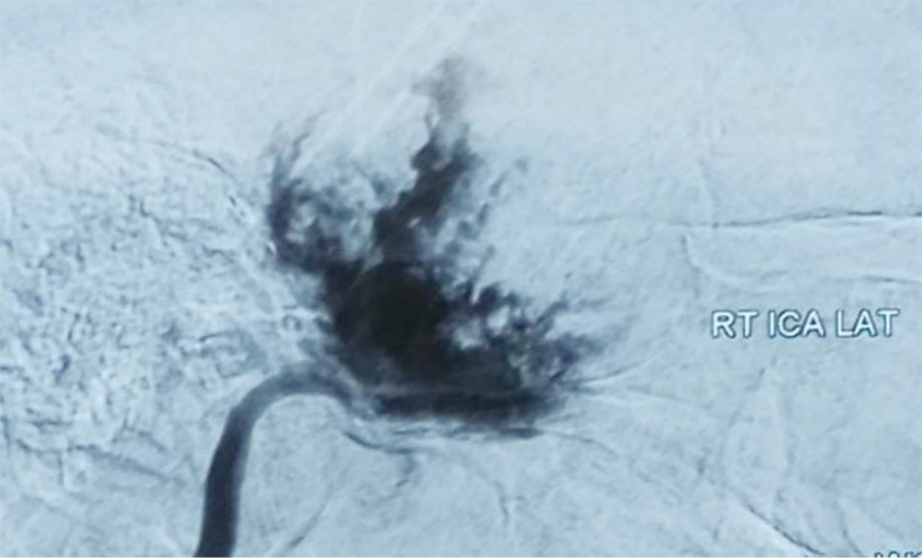
Digital Subtraction Angiography (DSA) showing blush after contrast injection in Right ICA (internal carotid artery). And no filling beyond cavernous segment.

Re-exploration was done in late April 2024 to excise the lesion. A highly vascular, thick-walled cavity was found along with hypoplastic right ICA preoperatively. A sudden gush of fresh sprouting bleed was encountered upon manipulating the cavity. Bleeding was controlled with temporary clip, cavity packed bleeding secured, bone removed, and procedure was abandoned for per-operative digital subtraction angiography (DSA) (Fig.3). There was no filling of right ICA beyond cavernous segment, right ophthalmic artery not visible. Right middle cerebral artery (MCA) segment M1 and posterior cerebral artery (PCA) segment P1 displaced upwards suggesting underlying mass. Diminutive caliber proximal portion of right ICA along with dilated right posterior communicating artery (P-com). Finding were suggestive of chronic compression on right ICA, probably underlying mass or stenosis. There was delayed filling of right MCA from left ICA injection and retrograde flow in right P-com from vertebral arteries injection.

**Fig.4:**
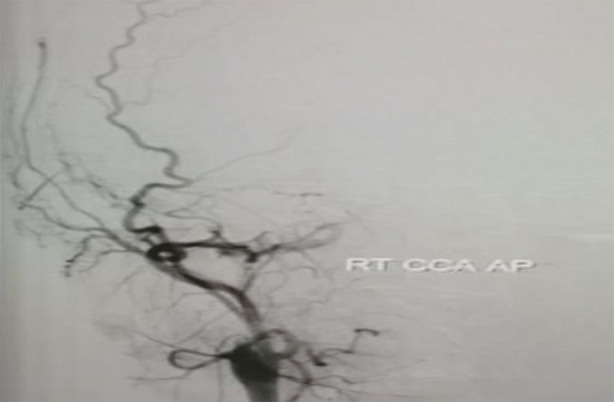
Post-operative Digital Subtraction Angiography (DSA) showing no distal filling in Right ICA (internal carotid artery) beyond cavernous segment.

Right ICA post cavernous segment was temporarily ligated and mass was dissected. Mass was greyish white extra-axial, firm in consistency, highly vascular encasing all the structures from the superior orbital fissure, adherent to sphenoid ridge, posterior clinoid process, middle cranial fossa, vessels were spared. Lesion was sent for biopsy, keeping the differential hemangioma in consideration. Microscopic inspection of specimen revealed a vascular lesion with multiple well-formed vessels. Hemangioma with OLIG2 and SSTR2 negative. Hence it was concluded to be a case of extra-axial, dural-based, skull base cavernous hemangioma.

Post-operatively patient remained neurologically on same status as preoperative. There was however CSF (cerebrospinal fluid) leak from wound which was managed with lumber drain kept for five days. Post-operative DSA was done, which professed no flow beyond right cavernous ICA.

## DISCUSSION

Intracranial cavernous hemangiomas are theorized to be formed from a thrombus or organized hematoma that ultimately undergo calcification in between the channels. They lack internal elastic lamina and smooth muscle, but can be ossified or calcified occasionally.^12^ They can grow in size exponentially, like our case and are called giant cavernous hemangiomas when >6cm.^7^ Growth potential depends on vessel ectasia along with angiogenesis.^13^

Al-Shahi et al. reported the common location of cavernous malformations in patients who presented with focal neurological deficit, as followings, lobar (34%) like our case in temporal lobe, brainstem (32%), cerebellum (21%), deep (13%), and multiple in 16%.^14^ Middle fossa hemangiomas associated with sellar region shows three patterns of growth: endophytic lateral, endophytic medial and exophytic pattern.^11^ Medial ones extends into sellar mimicking pituitary mass.^15^

Bradley et al. however reported most common symptoms as follows: seizures (37%), hemorrhage (36%), headache (23%) and focal neurological deficit (22%).^5^ Ene et al. reported the most common clinical finding, fits (40.6%), followed by symptomatic hemorrhage in 0.7-6.5%.^6^ Mustafa Caglar reported the case of cavernous hemangioma where patient only had headache.^16^ Our patient presented solely with focal neurological deficit, no signs and symptoms of hemorrhage, mainly because of the close proximity with cavernous sinus and orbit consistent with the present literature. Middle cranial fossa (MCF) hemangiomas that are located in cavernous sinus, sella para sellar region, sphenoid wing, or growing into MCF, presents with the symptoms of compression on nearby nerves i.e. CN III, IV, V, VI, may or may not occluding the ICAs and hypopituitarism.^5^,^7^

Typically appears well defined hypointense on T1, hyperintense signal on MRI is suggestive of bleed. Multiple flow voids intralesionally can be seen.^10^ Hyperintense on T2 and post contrast enhancement (typically peripheral) consistent with our imaging.^11^ Typical popcorn appearance, lobulated mass is also reported in literature. If bone erosion is present, T2 images give characteristic ‘salt and pepper’ appearance.^11^ Ours had contrast enhancement but no bone involvement. Digital subtraction angiography or CT or MRI angiography both are used to distinguish them from other vascular malformations i.e. AVMs, where both arterial feeder and venous drainage is seen.^16^ They also demonstrate the vascular anatomy, like abutting ICA or cavernous sinus or obscuring their flow.^10^

Histologically these appear like mulberry, irregular sinusoidal and vascular spaces.^8^ In literature both intra-axial (parenchymal based) and extra-axial (dural based) types are found.^8^ Former mostly manifest with hemorrhage and fits, later one however presents with headaches.^8^,^9^ However microscopically they both looks alike.^11^ But on gross inspection dural based are encapsulated and are attached to dura.^9^ They are found to adherent to cavernous sinus neurovascular structures when found in middle cranial fossa consistent with our per-op findings. Additionally, somatic GJA4 mutation is identified in extraxial cavernous hemangiomas.

These immensely vascular structure and prone to bleed upon handling surgically.^10^ While surgical excision remains the mainstay in management of cavernous hemangiomas but it carries a significant mortality and morbidity in terms of rebleed, fits, neurological deficit.^7^,^17^ Gross total resection can be achieved along with the ligation of feeding vessel if needed.^11^ We proceeded with temporary ligation of right ICA, to gain control on bleeding, followed by excision. Stereotactic radiosurgery (SRS) can be offered to selected patients with deep seated inaccessible lesions whilst the risk of hemorrhage is still there but it’s relatively very low.^4^ Gamma knife radiosurgery has shown significant size reduction and improvement in some not all neurological functions. Some prefer devascularizing the lesion prior to resection.^13^ Intraoperative frozen section can also be used to establish diagnosis when excessive bleeding is encountered during surgery and decision of total or subtotal resection can be made.^11^ Followed by SRS in cases of partial resection.^4^,^11^

## CONCLUSION

Cavernous hemangiomas are highly vascular structures and poses a great deal on morbidity and mortality, if they are not meticulously handled. MRI along with angiography and DSA can be used to establish vascular anatomy thereby aiding surgical decision. Gross total resection in safe hand is definitive plan of action followed by SRS or prior devascularization if required.

### Authors’ Contribution:

**ZMK** Concept and design of the work, critical review of the manuscript and supervision.

**HMQ & FIK:** Data acquisition and analysis, drafted manuscript.

**KA:** Data interpretation, critical review of the manuscript.

All authors agree to final approval of the version to be published be accountable for all aspects of the work.
